# Immunotherapeutic Strategies for Neuroblastoma: Present, Past and Future

**DOI:** 10.3390/vaccines9010043

**Published:** 2021-01-13

**Authors:** Fabio Morandi, Federica Sabatini, Marina Podestà, Irma Airoldi

**Affiliations:** Laboratorio Cellule Staminali Post-Natali e Terapie Cellulari, Istituto Giannina Gaslini (Istituto di Ricerca e Cura a Carattere Scientifico—IRCCS), Via G. Gaslini 5, 16147 Genova, Italy; fabiomorandi@gaslini.org (F.M.); federicasabatini@gaslini.org (F.S.); marinapodesta@gaslini.org (M.P.)

**Keywords:** neuroblastoma, immunotherapy, antibodies, CAR, NK, γδ T cells

## Abstract

Neuroblastoma is the most common extracranial pediatric solid tumor with a heterogeneous clinical course, ranging from spontaneous regression to metastatic disease and death, irrespective of intensive chemotherapeutic regimen. On the basis of several parameters, children affected by neuroblastoma are stratified into low, intermediate and high risk. At present, more than 50% of high-risk patients with metastatic spread display an overall poor long-term outcome also complicated by devastating long-term morbidities. Thus, novel and more effective therapies are desperately needed to improve lifespan of high-risk patients. In this regard, adoptive cell therapy holds great promise and several clinical trials are ongoing, demonstrating safety and tolerability, with no toxicities. Starting from the immunological and clinical features of neuroblastoma, we here discuss the immunotherapeutic approaches currently adopted for high-risk patients and different innovative therapeutic strategies currently under investigation. The latter are based on the infusion of natural killer (NK) cells, as support of consolidation therapy in addition to standard treatments, or chimeric antigen receptor (CAR) T cells directed against neuroblastoma associated antigens (e.g., disialoganglioside GD2). Finally, future perspectives of adoptive cell therapies represented by γδ T lymphocyes and CAR NK cells are envisaged.

## 1. General Features of Neuroblastoma

Neuroblastoma (NB) is the most widespread extracranial solid tumor in childhood [1,2] and the most common malignancy in the first year of life. The median age at diagnosis is 18 months and 90% of cases are diagnosed before the patient is 10 years of age [3]. It arises from the sympathetic nervous system, mainly from the adrenal medulla [4] and, in most cases, affects the adrenal glands or sympathetic ganglia [5]. At clinical presentation, NB is quite heterogeneous, ranging from asymptomatic tumors to diffuse metastases with systemic manifestations, thus reflecting on differences in outcome of patients that may evolve into spontaneous regression as well as into unfavorable progression, metastasis and death, irrespective of the intensive therapies adopted [6]. Metastases are present at diagnosis in about 50% of patients and mainly involve bone marrow (BM), bone and regional lymph nodes, while involvement of the central nervous system and lungs is rare, being present in less than 5% of metastatic patients at diagnosis [7,8]. In addition, extensive liver involvement may be observed in infants and causes liver disease, renal and lung dysfunction as a consequence of abdominal distention.

Diagnosis is based on a combination of laboratory tests, radiographic imaging and pathology, but many additional biological factors may be helpful to predict the clinical behavior in NB, including histologic and cytogenetic features, as well as molecular changes, in particular the amplification of the *MYCN* oncogene [9,10].

Remarkable efforts have been done by the International Neuroblastoma Risk Group (INRG) with the help of international groups, i.e., the Children’s Oncology Group and the International Society of Paediatric Oncology European Neuroblastoma, that created a cooperative task force in order to identify homogeneous risk groups before any treatment [11]. The extent of disease was determined by the presence or absence of image defined risk factors and/or metastatic disease at the time of diagnosis, defining disease stages as local (L1 and L2) or metastatic (M and MS). Furthermore, risk stratifications were defined including not only the stage, but different aspects of tumor biology [12] (Table 1).

The INRG collected data from over 8000 patients thanks to cooperation with different groups in North America, Europe and Japan and, when available, explored 35 different potential risk factors including prognostic factors such as age at diagnosis, pathology and genomic characterization (e.g., *MYCN* amplification and 11q status, cell ploidy and segmental chromosomal abnormalities), comparing these features to event-free and overall survival. Such efforts were of particular relevance since the precise risk stratification of patients were needed to guide therapy, improve the outcome for high-risk patients by intensification or changing treatment, and modify appropriately the chemotherapy for lower risk patients, with the aim of minimizing toxicity and late effects.

Thus, the INRG classified patients as low, intermediate or high risk: for the low and intermediate risk patients high overall survival greater than 90% has been achieved, while minimizing therapy [13,14,15]. By contrast, the high-risk patients show overall poor long-term outcome also complicated by devastating long-term morbidities, indicating that this group is specifically associated with chemo-resistance. The overall survival of high risk patients has improved over the past 20 years, from 29% for patients diagnosed from 1990 to 1994 to 50% for patients diagnosed from 2005 to 2010 [16,17]. Such results were presumably due to the intensification of therapy, myeloablation and immunotherapy, but prognosis of these patients still remains unsatisfactory. Nonetheless, patients with refractory or relapsed NB can rarely be cured and for this reason novel efficacious therapies are urgently needed.

## 2. Conventional Therapies for High Risk Patients

High-risk patients require intensive and complex therapies that include (i) the induction phase with multiple cycles of chemotherapy before surgery, (ii) a consolidation phase which may include myeloablation and autologous hematopoietic stem cell transplantation, local radiation and anti-disialoganglioside GD2 antibodies (Ab) and (iii) a maintenance phase with immunotherapy and/or differentiation agents [2].

The most widely used conventional cytotoxic chemotherapies are topotecan with either cyclophosphamide or temozolomide [18] or irinotecan and temozolomide [19,20,21] that may offer partial or even complete response with improvement in symptoms and quality of life, especially for low or intermediate risk patients.

At the end or soon after the end of induction chemotherapy, a surgical resection of the tumor mass, when possible, is applied in order to eliminate the remaining primary tumor.

Concerning the consolidation phase, it has been reported that myeloablation may significantly improve the outcome [22,23,24]. Although the autologous hematopoietic stem cell transplantation is commonly used, only marginal effects on event-free survival have been obtained and for this reason the optimal conditioning regimen is still under investigation. In this regard, long-term cures have been achieved by induction and stem-cell transplantation followed by anti-GD2 Ab therapy [25]. Alternatively, radiation therapy can be used locally.

The maintenance phase is generally composed of a combination of anti-GD2 Ab (that will be discussed in the following paragraph) and isotretinoin, known for its ability to induce differentiation and death in tumor cells, finally improving event-free survival in a randomized trial [23]. A phase III clinical trial is still active to test the side effects and efficacy of treating patients with NB (NCT01041638).

Another therapy is represented by the use of metaiodobenzylguanidine (MIBG), based on the finding that 90% of NB tumors express the norepinephrine transporter and therefore take up the sympathomimetic MIBG [26]. Clinical trials conducted in relapsed or refractory high-risk NB patients, using a high dose of ^131^I-MIBG as monotherapy or in combination with other agents, demonstrated a 30–40% response rate [27,28,29].

## 3. Immunological Features of NB

Similarly to other pediatric solid tumors, NB shows intrinsic immunological properties that render often ineffective therapies based on recruitment of cellular components of the immune system or the reactivation of them [30]. NB is poorly immunogenic as witnessed by (i) low expression of HLA class I molecules, (ii) defects in antigen-processing machinery, (iii) down-regulation of molecules that activate T and natural killer (NK) cells (i.e., PD-1L, NKG2DL, MIC-A and –B, ULBP-1, ULBP-2, ULBP-3 and DNAM-1 ligand), (iv) membrane expression and release of soluble HLA-G and –E, (v) release of exosomes containing immune-suppressive molecules (i.e., B7-H3) and (vi) secretion of immunomodulatory cytokines (e.g., IL-10 and TGF-β1) [31,32,33,34,35,36].

To complicate this picture, the tumor microenvironment is hypoxic thus contributing to a metabolic challenge for tumor cells themselves [37,38,39] and for infiltrating immune cells, leading to immune-suppression. The latter finding is supported by infiltration of tumor-associated macrophages (TAM) that are effective in paralyzing T cell responses, inducing T cell apoptosis through Fas–Fas ligand interactions and activating myeloid derived suppressor cells and regulatory T cells that, in turn, suppress active immune response [40,41,42]. Furthermore, NB cells express constitutively high levels of gangliosides which further contribute to the immune suppressive microenvironment. Of these, GD2 is one of the most studied surface antigens in NB also used for target therapies [43,44].

## 4. Immunotherapeutic Approaches for Neuroblastoma

### 4.1. Antibodies Targeting NB in Clinical Settings: the GD2 Disialoganglioside Prototype and Other Tumor-Associated Antigens

GD2 is an oncofetal antigen expressed during fetal development. It remains expressed after birth in neurons, peripheral nerves and skin melanocytes and it is consistently found in NB and osteosarcoma [43]. In these tumors, specific Ab targeting GD2 have been developed and used in clinical settings with encouraging results [25,45,46].

The effectiveness of GD2 Ab is related to different mechanisms as (i) direct induction of cell death, (ii) Fcγ receptor (FcγR)–mediated antibody-dependent cell-mediated cytotoxicity (ADCC) by immune cells such as NK, macrophages and neutrophils and (iii) complement-dependent cytotoxicity (CDC) [47,48,49,50]. Of note, these mechanisms of cytotoxicity may be highly potentiated by the use of immune stimulating cytokines or adoptive cell therapy. FDA approved two different GD2 Abs for clinical trials that are dinutuximab (Ch14.18, chimeric murine/human antibody to GD2) and naxitamab (hu3F8, a humanized murine antibody to GD2) [45,46,51], currently used in clinical trials in recruiting phase or still active.

Only one of these clinical trials reported data regarding progression-free survival (PFS) and overall survival (OS) comparing patients treated with irinotecan and temozolomide in combination with dinutuximab or temsirolimus [52]. Improved PFS and OS (76.5% and 88.2%, respectively) were observed in patients receiving dinutuximab compared to those treated with temsirolimus (24.7% and 64.7%, respectively)

**Figure 1:**
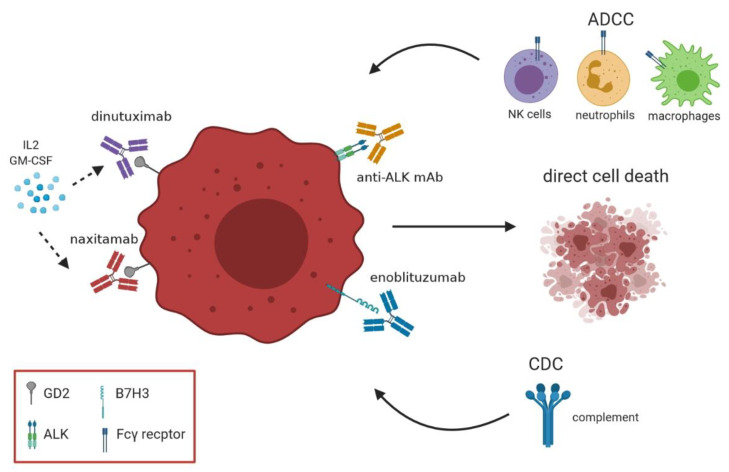
NB immunotherapy based on antibodies and mechanisms of action. The figure describes mechanisms of action of antibodies currently used in clinical settings. These antibodies specific for NB-associated antigens may induce direct cell death, complement-dependent cytotoxicity (CDC) and antibody-dependent cell-mediated cytotoxicity (ADCC). In addition, cytokines such as IL-2 and GM-CSF may increase the anti-tumor activity exerted by these antibodies.

Figure 1 summarizes the antibodies used for NB immunotherapy with corresponding antigens and mechanisms of action.

These Abs showed similar clinical impact and toxicities mainly manifested as neuropathic pain, fever and allergic reactions. However some differences have been reported probably due to their different half life [30,53].

Cytokines are the most factors used in combination with a GD2 antibody, especially IL-2 and granulocyte-macrophage colony stimulating factor (GM-CSF). IL-2 induces activation T and NK cells [54] and it was approved by FDA for treating adult tumors. In children with high-risk NB several phase I and II trials tested IL-2 as monotherapy, but no significant effective results have been reported [55,56,57]. Recently, Ladestein et al. demonstrated only marginal effects of dinutuximab beta in combination with IL-2. In fact, the 3-year event-free survival was 56% (95% CI 49–63) with dinutuximab beta (83 patients had an event) and 60% (53–66) with dinutuximab beta and subcutaneous IL-2 (80 patients had an event; *p* = 0.76) [25,48]. By contrast, IL-2 administration with alternating cycles of GM-CSF in combination with dinutuximab resulted in higher rates of event-free (66% versus 46%) and overall survival after 2 years, compared to standard therapy alone (86% versus 75%) [25,48]. GM-CSF is a myeloid growth factor that stimulates differentiation of progenitor cells into granulocytes and monocytes, and boosts immune responses [58]. A human recombinant GM-CSF, named LEUKINE^®^ (sargramostim), has been approved by FDA. Several clinical trials evaluating the role of GM-CSF combined with anti-GD2 in high-risk NB were conducted over the last decade (Table 2). Although this immunotherapy has shown substantial anti-neuroblastoma activity (NCT01757626) [51] even against minimal residual disease (NCT02100930), patients should be closely monitored for the onset of posterior reversible encephalopathy syndrome (NCT01183897) [26] (Table 2).

### 4.2. Other NB Associated Antigens as Targets for Antibody-Mediated Immunotherapy

Another target recently considered for development of therapeutic approaches against high risk patients is represented by anaplastic lymphoma kinase (ALK) that was found to be mutated in 9% of NB [59,60]. In 2009 the first clinical trial in pediatric patients with refractory solid tumors was conducted by the Children’s Oncology Group using an ALK inhibitor (i.e., crizotinib) providing discouraging results [61]. Afterwards, better results were obtained using other ALK inhibitor compounds, alone or in combination with chemotherapy, in solid tumors such as lung cancer, but some problems emerged due to secondary mutation and amplification of ALK and off-target mechanisms including activation of ‘bypass’ signaling pathways [62,63,64]. Recently, Sano R. et al. developed an Ab drug conjugate directly targeting ALK receptor, the CDX-0125-TEI, that exhibited cytotoxicity against both wild-type and mutated ALK in patient-derived xenografts [65] (Figure 1).

Finally, an additional interesting target for NB therapy is B7-H3 (CD276), a type I transmembrane glycoprotein molecule homogeneously expressed in both primary and metastatic NB (Figure 1) [62]. Loo D. et al. developed the anti B7-H3 Ab MGA27 that demonstrated to mediate potent cytotoxicity against a broad range of tumor cell types in xenograft models, while avoiding toxicities [66]. This antibody, later named as enoblituzumab, is currently in phase I trials for diverse solid tumors including refractory tumors and pediatric cancers.

## 5. Adoptive Cell Therapy Based on NK Cells

NK cells represent a subset of lymphocytes belonging to innate immune system, which originate in the BM and exert anti-tumor and anti-viral activities [67,68,69]. NK cell functions are regulated by the integration of multiple signals derived from activating and inhibitory receptors which bind specific ligands expressed on tumors and viral-infected cells. NK cells may be expanded ex vivo and stimulated by different cytokines, including IL-15, IL-12 and IL-18 [67,68,69]. Since NK cells do not cause graft versus host disease (GvHD), they represent an ideal source for allogeneic “off-the-shelf” cellular therapy. The development of therapeutic protocols for NB patients, based on the infusion of NK cells, arose from the success obtained in different clinical trials using monoclonal antibodies targeting NB. In these trials, the observed complete remission or stabilization of the disease was related to an increase of NK cell frequency and activity, including NK cell-mediated ADCC [70,71,72,73,74]. Of note, NK cells are known for their ability to target and lyse tumor cells lacking HLA-class I molecules, which represent the ligands of killer inhibitory receptors [75]. For this reason metastatic NB cells, which show low to absent HLA-class I expression [76], paralleled by the presence of different ligands for NK cell activating receptors [77], represent an ideal target for NK cell mediated lysis.

Different preclinical studies on NB have been described using NK cells. Castriconi et al. [78] analyzed the therapeutic effect of infusion of NK cells activated in vitro with IL-2 in a xenograft model of metastatic NB. SCID/NOD mice, inoculated intravenously with the HTLA-230 NB cell line, showed significant prolonged survival when injected with multiple NK cell doses soon after the inoculation of tumor cells. Such therapeutic effect was further increased by treatment with IL-2 and IL-15 that stimulated NK cells through up-regulation of the activating markers CD69 and CD25 as well as of NKp44 and DNAM-1 receptors [78]. Similarly, Liu and coworkers [79] tested NK cells, either from normal donors or NB patients, in SCID/NOD mice injected with CHLA-255-Fluc cells. At variance with the previous studies, NK cells were expanded in vitro, using a feeder composed of irradiated clinical-grade K562 cells expressing membrane-bound IL-21 (K562 mbIL-21), in the presence of IL-2. The resulting NK cells displayed high expression of DNAM-1, NKG2D, NKp46, CD56 and CD16, and efficiently lysed NB cell lines in vitro, through mechanisms such as (i) granzyme A and B release and (ii) induction of ADCC in the presence of anti-GD2 Ab. Preclinical studies highlighted a limited therapeutic effect of expanded NK cells alone, but a significant inhibition of tumor growth and a prolonged survival of mice when combined with the anti-GD2 ch14.18 Ab. These data suggested that the main therapeutic effect exerted by NK cells against NB in vivo was related to induction of ADCC rather than direct NK cell lysis [79]. The importance of this mechanism was confirmed in the study by Barry et al. that used primary NB xenografted in murine kidney, then analyzed for metastatic disease in the liver and BM. In this model, the administration of NK cells, expanded in vitro using K562 and IL-21, in combination with dinutuximab, not only reduced metastasis in the liver and BM, but also prolonged survival [80].

Starting from the concept that TGFβ is an immunosuppressive cytokine released by NB cells able to down-regulate activating receptors on NK cells and to inhibit the release of granzyme and perforin, Tran and coworkers performed preclinical studies using the TGFβR1 inhibitor galunisertib. They reported that mice, injected in the kidney with NB cell lines and treated with galunisertib plus expanded NK cells and dinutuximab, showed prolonged survival associated with reduction of tumor growth, as compared to mice treated with NK cells and dinutuximab alone. Thus, they paved the way to clinical application of this combined therapy for high-risk NB patients [81].

Clinical trials using NK cell infusions for NB patients are still ongoing (Table 3). A pilot trial was performed by Federico et al. [82] on NB patients that had undergone six different courses of induction chemotherapy, including cyclophosphamide and topotecan, or irinotecan and temozolomide, or ifosfamide, carboplatin and etoposide. Each group was treated with the anti-GD2 Ab hu14.18K322A, GM-CSF and IL2, in the presence or absence of haploidentical parental NK cell infusion. Such GMP-graded NK cells were isolated from leukapheresis by CliniMACS and infused at a median dose of 15.5 × 10^6^/Kg. NK cell infusion was safe and well tolerated, although toxicities related to chemotherapy, anti-GD2 or GM-CSF infusion were observed. The overall response rate of patients was 61.5%, with a complete response/very good partial response rate of 38.5%. More importantly, progression of the disease was never observed. However, the contribution of NK cells to therapeutic success in this limited study was not assessed [82]. A phase II clinical trial was performed in NB patients subjected to multiple cycles of induction chemotherapy with different drugs (including cyclophosphamide, topotecan, cisplatin, etoposide, doxorubicin and vincristine) and infused with the anti-GD2 Ab hu14.18K322A with GM-CSF and IL-2 [83]. Patients were treated, as consolidation therapy, with busulfan/melphalan plus infusion of autologous hematopoietic stem cells (collected in the induction therapy phase). In addition, some patients were treated with an experimental immunotherapy, composed of the hu14.18K322A Ab and GM-CSF in the presence or absence of GMP-graded haploidentical NK cells, which were purified using CliniMACS system and not expanded ex vivo. No difference in toxicities was observed between patients infused with or without NK cells. NK cells were still detected until 18 days post-infusion demonstrating an NK cell engraftment [83]. A longitudinal analysis of this clinical trial revealed that patients receiving haploidentical NK cell infusion had an increased NK cell count at day 7, and a decreased NK cell count at day 21 post-infusion [84]. However, donor NK cells infused at higher doses were still detected after 21 days. Such cells were able to respond to ex vivo stimulation with IL-15 and IL-2 and to lyse K562 cell line in vitro, thus suggesting that NK retained their cytotoxic function. Finally, patients with stable disease displayed a higher number of alloreactive NK cells than those with residual disease [84].

Modak and coworkers [85] performed a phase I clinical study on NB patients receiving an induction therapy with vincristine, cyclophosphamide and topotecan, and then different doses of haploidentical parental NK cells and the anti-GD2 murine Ab 3F8. In this study, NK cells were isolated by CliniMACS and then cultured overnight with IL-2 before being infused. Toxicities were almost related to the infusion of 3F8 Ab, whereas NK cell administration was safe and well tolerated. Moreover, they demonstrated that patients with detectable NK cells in the periphery at day 14 display a complete remission and, in general, patients who received a higher dose of NK cell infusion showed an improved progression-free survival compared to those receiving lower NK cell doses (*p* = 0.018) [85].

Of note, Heinze and coworkers [86] recently described different methods to expand NK cells in vitro for clinical use analyzing viability, purity and composition of NK cell preparations starting from normal donors’ buffy coat preparations. Either CD56^+^ enriched cells and CD3^+^/CD19^+^ depleted cells were cultured in media supplemented with different combinations of cytokines (i.e., IL-2+IL-15 or IL-15+IL-21). The authors concluded that the highest purity and expansion rate can be obtained starting from CD3^+^/CD19^+^ depleted cells using IL-15 and IL-21. These cells showed also increased cytotoxicity and degranulation, when cultured in the presence of NB cell lines, thus suggesting that these NK cell preparations could be useful to test in clinical trials [86].

Collectively, these studies suggested that NK cell infusion, as consolidation therapy, in NB patients is feasible and well tolerated. Therapeutic effects of NK cells against NB hold promise and have to be carefully evaluated in phase III randomized clinical trials. At present, no phase III/IV clinical trials using NK cell infusions are ongoing, whereas 20 phase I/II trials are registered (clinicaltrials.gov), and some of them completed (Table 3).

## 6. Adoptive Cell Therapy Based on NKT Cells

Another innate cytotoxic cell population, with intermediate features between NK and T cells, is represented by NKT cells which express an invariant TCRα chain, Vα24-Jα18, and are able to recognize self- and microbial-derived glycolipids presented by the monomorphic HLA class I-like molecule CD1d [87]. The contribution of NKT cells to anti-tumor responses has been demonstrated in different tumor models [88,89,90], as underlined by the finding that these cells are decreased in number and/or functionality in cancer patients [91,92,93]. NKT-based clinical trials are ongoing for several malignancies, and are based on the injection of alpha-Galactosylceramide (α-GalCer) or α-GalCer-pulsed APC to expand NKT cells in vivo. Alternatively, autologous NKT cells have been expanded ex vivo using IL-2 and anti-CD3 mAbs [94]. Recent evidence suggested that NKT cells may have anti-tumor activities in NB patients. Liu et al. [95] reported that NKT cell migration towards a co-culture of NB cells and monocytes was increased in hypoxic conditions, a typical feature of NB microenvironments. Indeed, hypoxia down-regulated the secretion of the tumor-associated chemokine CCL2 (which attracts NKT cells) by NB cells, whereas the chemokine CCL20 was increased upon co-culture of NB cells with monocytes. Accordingly, neutralizing monoclonal Abs against CCL20 abrogated NKT cell migration in hypoxic conditions [95]. The authors demonstrated that humanized NOD/SCID *Il2rg*^-/-^ (NSG) mice inoculated with NB cells in the renal capsule showed a high infiltration of human TAM in the tumor microenvironment, with a high percentage of M2 macrophages. Afterwards, they inoculated expanded NKT cells that highly infiltrated the tumors, but this feature was abrogated by the treatment of mice with anti-CCL2 and anti-CCL20 mAbs. Furthermore, expanded NKT cells, inoculated in preclinical model of metastatic NB, preferentially migrated in the hypoxic areas of metastasis in liver and BM, were inhibited by hypoxia and by contact with tumor cells [95]. To improve NKT cell functions, they transduced NKT cells with a cDNA encoding human IL-15 (NKT/IL-15), demonstrating that the latter cells were able to proliferate in hypoxic conditions in vitro, whereas parental NKT cells were inhibited in the same settings. Finally, they setup a metastatic model of NB in NSG and humanized NSG mice, showing that tumors developed more rapidly in the latter mice, due to the infiltration of human hematopoietic cells in the tumor. Such tumor-promoting effect was totally abrogated by inoculating NKT/IL-15 (but not NKT) and restored in the presence of CD1d blocking mAbs. These findings suggest that adoptive therapy with expanded NKT cells in combination with IL-15 may represent a therapeutic strategy for patients with metastatic NB [95]. So far, no active clinical trials based on NKT cells as therapy are present for NB patients.

## 7. CAR T Cells for Therapy of High Risk NB Patients

The use of T cells genetically modified to express a chimeric antigen receptor (CAR) is a new promising approach of adoptive cell therapy in cancer, combining the antigen specificity of a monoclonal Ab with the effector function and long-term persistence of T cells [96,97,98]. CAR provided success in treating B cell acute lymphoblastic leukemias, with limited clinical benefit in solid tumors, although more than 100 clinical studies have been developed. Indeed, the success of CAR T cell therapy in solid tumors relies on the ability of CAR T cells enter in the tumor site, overcome the immunosuppressive tumor microenvironment, and persist for a long period.

CAR is composed of different domains, including (i) the single-chain variable fragment (scFv) of tumor antigen-specific Ab, (ii) a hinge region, (iii) the trans-membrane domain of CD8α or CD28 molecules and (iv) the intracellular signaling region. Thus, T cells endowed with CAR showed an improved T-cell antigen recognition, T-cell activation and tumor cell lysis. CARs are classified as first, second and third generation on the basis of the presence of one, two or more T cell co-stimulatory molecules [99,100].

First generation CAR included activating/signaling domains of CD3ζ or FcγRIII molecules, whereas second generation CAR included co-stimulatory domains of other molecules, including CD28, ICOS, 4-1BB, OX-40 and CD27). The third generation CAR are endowed with co-stimulatory domains in tandem (i.e., CD28 in combination with 4-1BB), which may increase T cell expansion and anti-tumor functions [101].

FDA approved two different products for CAR T cell therapy in 2017, named Kymriah^®^ (Novartis) and Yescarta^®^ (Kite/Gilead), for the treatment of hematological malignancies [102].

The most common side effects of CAR αβ T cells are cytokine release syndrome (CRS) and GvHD [103]. Of note, to prevent unexpected T-cell–related toxicity such as CRS, an inducible caspase-9 (iC9) gene was introduced in frame with the TCR, thus allowing the prompt elimination of genetically modified T cells by the use of chemical induction of dimerization (CID) drug, AP1903 [104,105].

Two clinical studies have been conducted with first generation CAR T cells in patients with NB [106,107]: in the first GD2 CAR T cells were well tolerated, but only one of six patients had a partial response, in the second five of 11 patients with active disease showed tumor responses and three of them had complete responses. Nonetheless, the response rates in patients with NB remain significantly lower than those observed in patients with acute lymphoblastic leukemia treated with CD19-CAR constructed with the same approach [108]. More recently, Heczey A et al. implemented a third-generation GD2 CAR in which the specific scFv was derived from the murine 14.G2a mAb coupled with the ξ-chain endodomain and two costimulatory endodomains in tandem (CD28 and OX40) [109]. CAR T cells, administered in combination with lymphodepletion to patients with NB, were well tolerated and promoted some objective clinical responses [107,109,110]. Afterwards, Quintarelli et al. [111] reported that insertion of 4-1BB costimulatory domain, in the place of OX40 in a third generation CAR, significantly improved the anti-tumor efficacy of GD2 CAR. The choice of 4-1BB signaling was particularly effective in terms of T cell activation and persistence, control of tumor cell growth and T cell exhaustion [111].

Finally, Chen and coworkers incorporated IL-15 and iC9 within the GD2 CAR construct and demonstrated that transduced T cells enriched in central memory/stem cell–like cells, expressed PD-1 at low level, promoted superior antitumor activity, expansion and survival in vitro and in vivo [112].

Several clinical trials are in progress (Table 4), including those that evaluate CAR T cells that target CD171 [113] in patients with NB and ganglioneuroblastoma (NCT02311621) or B7H3 (NCT04483778) in relapsed/refractory pediatric solid tumors. Many others continue to explore GD2–CAR T cells for NB and include NCT02761915, NCT03373097, NCT03721068, NCT03635632, NCT01822652 and NCT01953900. In addition, several studies have addressed strategies to implement the efficacy of CAR T cell therapies in NB patients [114].

**Figure 2:**
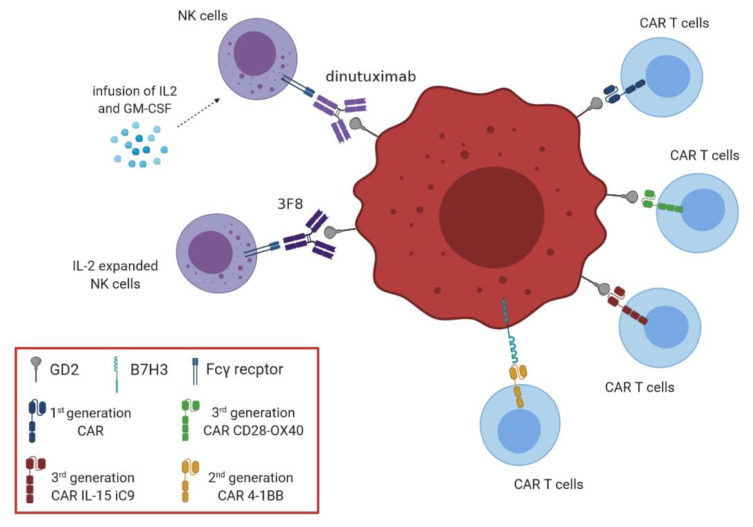
Current adoptive cell therapies in the clinical setting of neuroblastoma (NB) treatment. This figure describes different adoptive cell therapy approaches currently investigated for immunotherapy of high-risk NB patients. This includes CAR T cells and NK cells as adjuvant therapy, used alone or in combination with cytokines. Cytokines may also be used to expand NK cells before infusion.

Figure 2 summarizes current approaches for adoptive cell therapy in the clinical setting of NB.

## 8. Future Prospects: CAR NK Cells and γδ T Cells

γδ T cells and NK cells may represent an ideal source for adoptive cell therapy due to some shared features. Both cell types recognize and kill tumor cells irrespective of the expression of a single tumor-associated antigen, thus avoiding tumor immune escape related to single antigen loss [67,68,69,94,114,115]. In addition, γδ T and NK cells act through MHC-independent recognition of target cells, thus reducing the risk of alloreactivity and GvHD [116] and are able to provide immediate release of effector cytokines in different tissues. Of note γδ T cells show a natural tissue tropism which provide a migratory advantage over αβ T cells, leading to a superior ability to infiltrate in hypoxic tumors [117,118]. Finally, γδ T cells can interact with APC and other immune cells, and also act as APCs by priming the antigens for αβ T cells, thus orchestrating the anti-tumor immune responses [119,120].

Taken together, these features render NK and γδ T cells an optimal source for immunotherapy, and different genetic engineering strategies have been developed to enhance and redirect their cytotoxicity toward tumor antigens as well as to improve their in vivo or ex vivo expansion that is crucial for their broad clinical application [121].

In this regard, although γδ T cells may be difficult to expand efficiently ex vivo, sufficient numbers for adoptive transfer immunotherapy especially for pediatric patients are easily obtained. For example, the Vγ9Vδ2 population may be efficiently expanded using zoledronic acid and IL-2 both ex vivo and in vivo starting from peripheral blood mononuclear cells [99]. γδ T cell infusion proved to be safe and showed promising results in recent clinical trials. Indeed, ongoing studies are addressing the increase of γδ T cell survival and potency for clinical purposes [122].

Although CAR γδ T cells have shown preclinical efficacy, their optimization and the choice of the best co-stimulatory domains are still open [123]. γδ T cells express a wide panel of co-stimulatory molecules, including CD28, CD27 and 4-1BB that enhance γδ T cell survival and proliferation. Thus, the interaction between signals derived from γδ TCR and these molecules should be studied in detail to design more suitable CAR constructs [124].

The clinical efficacy of CAR T cells in solid tumors may be inhibited by immune suppressive conditions in the tumor microenvironment [123]. In this regard, γδ T cells may be resistant to immune-suppression due to the expression of peculiar receptors. In addition, the addition of treatments targeting the stroma and checkpoint inhibitors may improve the treatment efficacy in solid tumors.

Polito et al. [125] recently described an efficient method to produce allogeneic third-party and ready-to-use γδ CAR T cells. They expanded memory Vδ1 subpopulation using engineered APCs expressing CD86, 4-1BBL, CD40L and the CMV-antigen-pp65, obtaining polyclonal γδ T cells expressing activation and memory markers, with potent anti-tumor activity in vitro and in vivo with no sign of alloreactivity. Finally, the authors engineered these cells with a third generation anti-GD2 CAR (GD2.CD28.4-1BBζ), further enhancing anti-NB cytotoxicity.

A handful of clinical trials using γδ T cells for cancer treatment have been performed, several are in progress and include hematological malignancies (NCT02656147, NCT03533816, NCT04008381 and NCT03885076), glioblastoma (NCT04165941), hepatocellular carcinoma (NCT00562666, NCT02425735 and NCT04518774), renal carcinoma (NCT00582790), breast cancer (NCT02418481 and NCT03183206), prostate cancer (NCT00582556), lung cancer (NCT0318323, NCT02425748, NCT00588913) and pancreatic cancer (NCT03180437). These clinical studies revealed that adoptive transfer of γδ T cells is safe and feasible [126] and, in most cases, objective responses were observed.

NK cells represent another ideal source for allogeneic “off-the-shelf” cellular therapy, since such cells (i) do not cause GvHD, (ii) lack in vivo clonal expansion and (iii) display a limited persistence. These features reduce the risk of CRS, a life-threatening toxicity observed in several CAR αβ T cell trials [127]. However, a limitation of this therapeutic approach may be related to a different persistence between CAR T and CAR NK cells according to disease, the tumor burden, lymphodepletion settings and other factors (i.e., the potency of the administered T cells and CAR construct). Thus, some strategies have been developed to overcome this issue, including the co-transfection of stimulatory cytokines (e.g., IL-15) and multiple administration of CAR cells at different time points.

First, second and third generation CAR NK cells have been generated, using lentiviral vectors, to target tumor antigens such as CD19 [128,129], CD20 [128], CD33 [130], CD7 [131], CD22 [132], ErbB2 [133] and EGFR [134,135]. Such CARs contained the scFv region of a tumor antigen-specific Ab combined with (i) enhancers like Kozak sequence (HU 2020), (ii) signaling domains including 4-1BB [129,130,136,137] and/or CD28 [130,133,134,135,136,138], (iii) CD8α hinge region [137,138], (iv) myc tag sequence [139] and (v) CD3ζ chain of TCR complex [128,129,133,134,135,138,140].

Esser [141] and Romanski [142] used amphotropic retroviral vectors to generate CAR NK cells. The construct was composed of scFv of ch14.18 anti-GD2 chimeric Ab or of anti-CD19 Ab, respectively, combined with IgVH signal peptide, myc tag, the hinge region of CD8α and trans-membrane/intracellular domains of CD3ζ chain. Such CAR NK cells recognized and eliminated GD2 expressing cells which were resistant to parental NK-92 cells. In addition, the authors observed increased killing of NK-sensitive tumor cell lines using retargeted NK-92 cells. Such lysis was strictly dependent on specific recognition of the target antigen.

The potency of this therapeutic approach was further implemented by Kailayangiri et al. [143] by the integration of co-stimulatory molecules (i.e., trans-membrane domain of CD28 and signaling domains of 4-1BB and CD3ζ) and by extended cytokine support during NK cell expansion in vitro. However, the in vivo response was low, due to the immunosuppressive activities of HLA-G molecule.

To date, only a few phase I/II clinical trials are in recruiting status for NB therapy (NCT03940820, NCT02839954 and NCT03941457), but many others are in progress for different diseases including Covid-19 (NCT04324996), solid tumors (NCT03940820, NCT02839954, NCT03941457), glioblastoma (NCT03383978) and hematological malignancies (NCT04008394, NCT02274584, NCT04526834, NCT04538599, NCT03940833, NCT04623944, NCT03056339, NCT04004637, NCT03049449, NCT04033302, NCT04288726, NCT04538599 and NCT04008394).

## 9. Conclusions

In the last 20 years several biological parameters that impact on the course of NB and on patients’ survival have been identified. These are of particular relevance for the best risk stratification, thus identifying high risk patients who need more aggressive treatments to improve their clinical outcome. In this line, a plethora of therapeutic strategies have been setup and applied in preclinical and clinical settings for patients with refractory or relapsed tumors. Nonetheless, these patients can rarely be cured, and for this reason innovative and more effective protocols are urgently needed.

Anti-GD2 Abs represent the first immunotherapy applied in NB which greatly increase overall and progression-free survival of children, especially when used in combination with cytokines, such as IL-2 and GM-CSF.

Recent advances have been achieved using NK cells as adoptive cell therapy in support of consolidation phase, and infusion of CAR T cells. In this regard, many phase I/II clinical trials have been completed, some are still ongoing, demonstrating that adoptive cell therapy is safe, well tolerated and without relevant toxicities. However, efficacy of both adoptive cellular therapeutic strategies has to be confirmed in future phase III/IV studies. Of note, NK cells will be used as adjuvant therapy after at least one cycle of standard therapy plus anti-GD2 mAbs whereas CAR T cells will be infused in patients with relapse or refractory to conventional treatments.

Future perspectives of adoptive cell therapies for high-risk NB patients are represented by the use of γδ T cells and by the generation of novel CAR cells obtained by the transfection of NK and γδ T cells. Undoubtedly, the peculiar advantage of these cells is the generation of “off the shelf” therapies avoiding a strict selection of the donors irrespective of the recipients, with no risk of GvHD. To date, few phase I/II on NB patients are ongoing, however promising results have been obtained in other malignancies, demonstrating the safety of these therapeutic approaches.

## Figures and Tables

**Table 1:** International Neuroblastoma Risk Group Pretreatment Classification Scheme.

NRG Stage	Age (Months)	Histologic Category	Grade of Tumor Differentiation	MYCN	11q Aberration	Ploidy	Pretreatment Risk Group
L1/L2		GN maturing, GNB intermixed					A (very low)
L1		Any, except GN maturing or GNB intermixed		NA			B (very low)
				Amplified			K (high)
L2	<18	Any, except GN maturing or GNB intermixed		NA	No		D (low)
					Yes		G (intermediate)
	≥18	GNB nodular, neuroblastoma	Differentiating	NA	No		E (low)
					Yes		H (intermediate)
			Poorly differentiated or undifferentiated	NA			H (intermediate)
				Amplified			N (high)
M	<18			NA		Hyperdiploid	F (low)
	<12			NA		Diploid	I (intermediate)
	12 < 18			NA		Diploid	J (intermediate)
	<18			Amplified			O (high)
	≥18						P (high)
MS	<18			NA	No		C (very low)
					Yes		Q (high)
				Amplified			R (high)

Abbreviations: GN = ganglioneuroma; GNB = ganglioneuroblastoma; INRG = International Neuroblastoma Risk Group; NA = not amplified.

**Table 2:** Clinical trials using granulocyte-macrophage colony stimulating factor (GM-CSF) and anti-GD2 in the last ten years.

Identifier	Study Title	Phase	Start Date	Patients enrolled	Status	Primary Aims and References
NCT01183897	3F8/GM-CSF immunotherapy plus 13-cis-retinoic acid for primary refractory Neuroblastoma in bone marrow	II	2010	31	Completed	To find out what effects, good and/or bad, the combination of 3F8 and GM-CSF had on the patient and the cancer Another purpose was to see if high-dose 3F8 combined with GM-CSF is better than standard dose 3F8 in treating neuroblastoma Kushner BH et al. Cancer. 2013.
NCT01183429	3F8/GM-CSF immunotherapy plus 13-cis-retinoic acid for consolidation of first remission after non-myeloablative therapy in patients with high-risk neuroblastoma	II	2010	39	Completed	
NCT01183416	High-Dose 3F8/GM-CSF immunotherapy plus 13-Cis-Retinoic acid for consolidation of first remission after myeloablative therapy and autologous stem-cell transplantation	II	2010	4	Completed	
NCT01041638	Monoclonal antibody Ch14.18, sargramostim, aldesleukin and isotretinoin after autologous stem cell transplant in treating patients with neuroblastoma	III	2010	105	Active, not recruiting	To study the side effects of giving monoclonal antibody Ch14.18 together with sargramostim, aldesleukin and isotretinoin after autologous stem cell transplant in treating patients with neuroblastoma
NCT01334515	Biological therapy, sargramostim and isotretinoinin treating patients with relapsed or refractory neuroblastoma	II	2011	52	Completed	To analyse how well hu14.18-interleukin-2 (IL2) fusion protein works when given together with sargramostim and isotretinoin in treating patients with relapsed or refractory neuroblastoma (Shusteman S et al. Clin Cancer Res. 2019)
NCT01757626	Combination therapy of antibody Hu3F8 with GM-CSF in patients with relapsed/refractory high-risk neuroblastoma	I/II	2012	224	Recruiting	To find out if an antibody called Humanized 3F8 (Hu3F8) combined with granulocyte-macrophage colony stimulating factor is safe for treating neuroblastoma (Kushner BH et al. JAMA Oncol. 2018).
NCT01592045	ch14.18 Pharmacokinetic study in high-risk neuroblastoma	I/II	2012	28	Completed	To compare the pharmacokinetics (blood levels) and safety of chimeric 14.18 manufactured by two independent drug makers
NCT01767194	Irinotecan hydrochloride and temozolomide with temsirolimus or dinutuximab in treating younger patients with refractory or relapsed neuroblastoma	II	2013	73	Completed	To investigate how well irinotecan hydrochloride and temozolomide with temsirolimus or dinutuximab work in treating younger patients with neuroblastoma that has returned or does not respond to treatment. Mody R et al. Lancet Oncol. 2017.
NCT02100930	Anti-GD2 3F8 monoclonal antibody and GM-CSF for high-risk neuroblastoma	NA	2014	69	Completed	To supply an experimental combination of drugs called 3F8 and sargramostim to patients with high-risk neuroblastoma who may benefit from treatment.
NCT02173093	Activated T cells armed with GD2 bispecific antibody in children and young adults with neuroblastoma and osteosarcoma	I/II	2014	40	Recruiting	To study the side effects and best dose of activated T cells armed with GD2 bispecific antibody and how well they work in treating patients with neuroblastoma, osteosarcoma and other GD2-positive solid tumors.
NCT02130869	A pilot study of immunotherapy including haploidentical NK cell infusion following CD133+ positively-selected autologous hematopoietic stem cells in children with high risk solid tumors or lymphomas	I	2014	8	Completed	In NB, to establish NK cell engraftment in patients receiving high-dose chemotherapy, stem cell infusion, hu14.18K322A, IL-2, haploidentical natural killer cell infusion, G-CSF and GM-CSF.
NCT02786719	High-risk neuroblastoma chemotherapy without G-CSF	NA	2016	13	Completed	Whittle SB et al. Blood Cancer. 2020
NCT03363373	Naxitamab for high-risk neuroblastoma patients with primary refractory disease or incomplete response to salvage treatment in bone and/or bone marrow	II	2017	95	Recruiting	To define the effects of naxitamab and GM-CSF in children diagnosed with high-risk neuroblastoma with primary refractory disease or incomplete response to salvage treatment
NCT03033303	A study of the effect of Hu3F8/GM-CSF immunotherapy plus isotretinoin in patients in first remission of high-risk neuroblastoma	II	2017	59	Recruiting	To test the combined effects of Humanized 3F8 in combination with granulocyte-macrophage colony stimulating factor
NCT03189706	Study of chemoimmunotherapy for high-risk neuroblastoma	I	2017	62	Recruiting	To test the efficacy and safety of Hu3F8 combined with the chemotherapy drugs irinotecan and temozolomide or GM-CSF
NCT04211675	NK cells infusions with irinotecan, temozolomide, and dinutuximab	I/II	2019	31	Not yet recruiting	Phase 1: to assess the safety and tolerability of autologous expanded NK cells in combination with irinotecan, temozolomide and dinituximab. Phase 2: to estimate the response to treatment
NCT03794349	Irinotecan hydrochloride, temozolomide, and dinutuximab with or without eflornithine in treating patients with relapsed or refractory neuroblastoma	II	2019	95	Recruiting	To study how well irinotecan hydrochloride, temozolomide and dinutuximab work with or without eflornithine in patients with relapsed or refractory neuroblastoma
NCT04560166	IT with or without naxitamab and GM CSF in patients with high-risk neuroblastoma	III	2020	117	Not yet recruiting	This is an open label, randomized, controlled, multicenter phase 3 trial, in patients ≥ 12 months of age with high-risk NB with primary refractory disease or in first relapse
NCT04385277	Treatment with dinutuximab, sargramostim (GM-CSF), and isotretinoin in combination with irinotecan and temozolomide after intensive therapy for people with high-risk neuroblastoma (NBL)	II	2020	45	Not yet recruiting	Safety of dinutuximab, GM-CSF and isotretinoin in combination with irinotecan and temozolomide, in high-risk neuroblastoma patients after consolidation therapy

Abbreviations: 3F8, a murine immunoglobulin 3 monoclonal antibody specific for disialoganglioside; Hu3F8, Humanized murine IgG3 anti-GD2 antibody m3F8; GM-CSF, subcutaneous granulocyte-macrophage-colony-stimulating factor.

**Table 3:** Phase I/II Clinical trials of NK Immunotherapy.

Identifier	Study Title	Phase	Start Date	Patients Enrolled	Status	Primary Aims and References
NCT01386619	NK DLI in patients after human leukocyte antigen (HLA)-haploidentical hematopoietic stem cell transplantation (HSCT)	I/II	2004	15	Completed	To analyse the feasibility of expanded NK-cell DLI production and their safety, by evaluating regards transfusion associated adverse events and the absence of acute graft-versus-host disease 30 days after the last NK DLI infusion. The efficacy of NK DLI infusions has been also assessed by evaluating the rates of overall and disease free survival and the rate of disease relapse compared to patients treated with haploidentical HSCT without NK DLI infusions.
NCT00569283	Donor natural killer cell infusion in preventing relapse or graft failure in patients who have undergone donor bone marrow transplant	I	2007	18	Completed	To evaluate the safety of donor natural killer (NK) cells, given as a single intravenous infusion in patients that underwent HLA-haploidentical familial donor bone marrow transplantation and to determine the maximum tolerated dose of donor NK cells infusion. The effectiveness of donor NK cell infusion was evaluated as ability to prevent tumor relapse and graft failure.
NCT00877110	Anti-GD2 3F8 antibody and allogeneic natural killer cells for high-risk neuroblastoma	I	2009	71	Completed	To assess the feasibility and safety of administering allogeneic haploidentical NK infusions with mAb 3F8 in patients with high-risk NB. Moreover, the efficacy of allogeneic NK infusions plus 3F8 was evaluated as anti-tumor activity. The impact of KIR/HLA immunogenetics and CD16 polymorphism on disease response to NK/3F8 was also evaluated.
NCT01462396	Allogeneic stem cell transplantation for advanced neuroblastoma using MHC mismatched related donors	I	2011	4	Completed	To evaluate the safety of a fludarabine based reduced intensity conditioning regimen and CD34^+^ stem cell selected mis-matched, related, allogeneic transplant in patients with relapsed/refractory neuroblastoma, by monitoring mortality, toxicity, acute and chronic graft versus host disease and engraftment rate. Anti-tumor effect has been also evaluated.
NCT01287104	A phase I study of NK cell infusion following allogeneic peripheral blood stem cell transplantation from related or matched unrelated donors in pediatric patients with solid tumors and leukemias	I	2011	34	Completed	To assess the feasibility and toxicity of infusing escalating doses of donor-derived activated NK cell donor lymphocyte infusions (NK-DLI) following human leukocyte antigen (HLA)-matched T cell depleted (TCD) peripheral blood stem cell transplant (PBSCT) in patients with metastatic or recurrent pediatric solid tumors and high risk leukemias who have unrelated donors or related donors. In addition, donor engraftment and acute graft versus host disease have been evaluated. Shah NN et al. Blood. 2015.
NCT01386619	NK DLI in patients after human leukocyte antigen (HLA)-haploidentical hematopoietic stem cell transplantation (HSCT)	I/II	2011	15	Completed	To evaluate the feasibility of NK-DLI production. The safety of NK DLI Infusion has been evaluated as transfusion associated adverse events (fever, fall in blood pressure, transfusion site reactions, etc) at the time of NK DLI infusion. The primary long-term safety measure is the absence of acute graft-versus-host disease 30 days after the last NK DLI infusion. The efficacy of NK DLI infusions has been assessed as rate of overall and disease free survival and disease relapse.
NCT01576692	Combination chemotherapy, monoclonal antibody, and natural killer cells in treating young patients with recurrent or refractory neuroblastoma	I	2012	34	Completed	To evaluate the toxicity associated with humanized anti-GD2 antibody/chemotherapy associated or not with NK cells infusion. Clinical outcome was measured as response to therapy using response evaluation criteria in solid tumors, clearing of bone marrow and improvement in MIBG scans. Event-free and overall survival have been also analyzed.
NCT01701479	Long term continuous infusion ch14.18/CHO plus s.c. aldesleukin (IL-2)	I/II	2012	288	Active, not recruiting	To find a way of giving ch14.18/CHO, in combination with subcutaneous aldesleukin (IL-2) and oral isotretinoin (13-cis-RA), to children and young people with primary refractory or relapsed neuroblastoma without intravenous morphine.
NCT01875601	NK white blood cells and interleukin in children and young adults with advanced solid tumors	I	2013	16	Completed	To evaluate the feasibility of harvesting and expanding activated NK cells and to assess the toxicity of infusing escalating doses of activated NK cells following lymphodepleting chemotherapy with or without escalating doses of rhIL15 in pediatric patients with refractory malignant solid tumors. Kontny HU et al. Cell Death Differ. 2001. Yu AL et al. N Engl J Med. 2010. Dudley ME et al. Nat Rev Cancer. 2003.
NCT01857934	Therapy for children with advanced stage neuroblastoma	II	2013	153	Active, not recruiting	To analyze event-free survival of patients with newly diagnosed high-risk NB treated with hu14.18K322A in addition to standard treatment. In addition, the tolerability of hu14.18K322A with allogeneic natural killer (NK) cells from an acceptable parent, in the immediate post-transplant period will be evaluated. Tolerability of hu14.18K322A with interleukin-2 and GM-CSF as treatment for minimal residual disease (MRD) will be assessed. Nguyen R et al. J Immunother Cancer. 2020.
NCT01807468	Haploidentical stem cell transplantation and NK cell therapy in patients with high-risk solid tumors	II	2013	12	Active, not recruiting	To evaluate feasibility and efficacy of haploidentical stem cell transplantation followed (or not) by NK cell infusion in patients with high-risk solid tumors who failed after tandem high-dose chemotherapy and autologous stem cell transplantation.
NCT02130869	A pilot study of immunotherapy including haploidentical NK cell infusion following CD133+positively-selected autologous hematopoietic stem cells in children with high risk solid tumors or lymphomas	I	2014	8	Completed	To investigate the addition of haploidentical natural killer (NK) cell infusion to high dose chemotherapy and autologous stem cell transplantation in children with high-risk solid tumors. In patients with neuroblastoma, the anti-GD2 antibody hu14.18K322A has been also given. Survival of children treated with this approach has been analyzed.
NCT02100891	Phase 2 STIR trial: Haploidentical transplant and donor natural killer cells for solid tumors	II	2014	15	Active, not recruiting	In this study, patients with high-risk solid tumors (Ewings sarcoma, neuroblastoma and rhabdomyosarcoma) underwent haploidentical hematopoietic cell transplantation (HCT) followed by an early, post-transplant infusion of donor natural killer (NK) cells. Safety and efficacy will be analyzed. Efficacy will be evaluated as complete (CR) and partial (PR) response and stable disease (SD). Hattinger CM et al. Expert Opin Emerg Drugs. 2019.
NCT02573896	Immunotherapy of relapsed refractory neuroblastoma with expanded NK cells	I	2015	24	Recruiting	To determine the maximum tolerated dose of autologous expanded natural killer (NK) cells when combined with standard dosing of ch14.18 and will assess the feasibility of adding lenalidomide at the recommended Phase II dose of the expanded NK cells with ch14.18, for treatment of children with refractory or recurrent neuroblastoma.
NCT02650648	Humanized anti-GD2 antibody Hu3F8 and allogeneic natural killer cells for high-risk neuroblastoma	I	2016	85	Active, not recruiting	To see if it is safe and feasible to give the participant cyclophosphamide, natural killer (NK) cells and Hu3F8 antibody as a treatment for neuroblastoma.
NCT02508038	Alpha/Beta CD19+Depleted haploidentical transplantation+zometa for pediatric hematologic malignancies and solid tumors	I	2016	22	Recruiting	To study safety of transplantation with a haploidentical donor peripheral blood stem cell graft depleted of TCRαβ^+^ and CD19^+^ cells in conjunction with the immunomodulating drug Zoledronate, given in the post-transplant period to treat pediatric patients with relapsed or refractory hematologic malignancies or high risk solid tumors.
NCT03242603	Immunotherapy of Neuroblastoma Patients Using a Combination of Anti-GD2 and NK Cells	I/II	2017	5	Unknown	To measure tumor response after infusion of expanded activated haploidentical NK cells with anti-GD2. Disease response will be defined as complete response/remission (CR), partial response (PR), minor response, stable disease (SD), or progressive disease (PD).
NCT03209869	Treatment of relapsed or refractory neuroblastoma with expanded haploidentical NK cells and Hu14.18-IL2	I	2018	6	Suspended	Patients with relapsed or refractory neuroblastoma received ex vivo expanded and activated natural killer (NK) cells from haploidentical donor with the immunocytokine, hu14.18-IL2. Safety has been evaluated as adverse event and GvHD rate and efficacy was evaluated as progression-free and overall survival and/or objective tumor response.
NCT04211675	NK cells infusions with irinotecan, temozolomide, and dinutuximab	I/II	2019	31	Not recruiting	Phase 1: to assess the safety and tolerability of autologous expanded NK cells in combination with irinotecan, temozolomide and dinituximab. Phase 2: to estimate the response to treatment.

**Table 4:** Ongoing clinical trials using CAR T cells.

Identifier	Study Title	Phase	Start Date	Patients Enrolled	Status	Primary Aims
NCT00085930	Blood T cells and EBV specific CTLs expressing GD2 specific chimeric T cell receptors to neuroblastoma patients	I	2004	19	Active, not recruiting	To evaluate the safety of escalating doses of 14g2a.zeta chimeric receptor transduced autologous EBV specific cytotoxic T-lymphocytes and 14g2a.zeta transduced autologous peripheral blood T cells
NCT01822652	3rd generation GD-2 chimeric antigen receptor and iCaspase suicide safety switch, neuroblastoma, GRAIN	I	2013	11	Active, not recruiting	To define the dose limiting toxicities at six weeks post T cell infusion
NCT01953900	iC9-GD2-CAR-VZV-CTLs/refractory or metastatic GD2-positive sarcoma and neuroblastoma	I	2013	26	Active, not recruiting	To evaluate the safety and feasibility of intravenous injections of autologous iC9-GD2-CAR-VZV-CTLs in combination with VZV vaccination in patients with advanced GD2-positive sarcomas or neuroblastoma
NCT02311621	Engineered neuroblastoma cellular immunotherapy (ENCIT)-01	I	2014	40	Recruiting	Patients will be evaluated through day 28 for occurrence of dose limiting toxicity
NCT02761915	A cancer research UK trial of anti-GD2 T-cells (1RG-CART)	I	2016	27	Recruiting	To evaluate (i) the feasibility of 1RG-CART therapy in patients with relapsed or refractory neuroblastoma, (ii) safety and tolerability of 1RG-CART therapy and (iii) to assess the incidence, severity and causality of adverse events to 1RG-CART and/or the lymphodepleting regimen
NCT02919046	Study evaluating the efficacy and safety with CAR-T for relapsed or refractory neuroblastoma in children	NA	2016	22	Unknown	To establish the overall efficiency of patients with neuroblastoma after autologous CAR-T cell therapy
NCT02765243	Anti-GD2 4th generation CART cells targeting refractory and/or recurrent neuroblastoma	I	2016	20	Recruiting	To determine the toxicity profile of the 4SCAR-GD2-modified T cells with common toxicity criteria for adverse effects
NCT03373097	Anti-GD2 CAR T cells in pediatric patients affected by high risk and/or relapsed/refractory neuroblastoma or other GD2-positive solid tumors	I/II	2017	42	Recruiting	Phase I—Identification of the dose limiting toxicity Phase II—Assessment of Antitumor effect and best overall response
NCT03294954	GD2 specific CAR and interleukin-15 expressing autologous NKT cells to treat children with neuroblastoma	I	2017	24	Recruiting	To define the maximum tolerated dose of autologous NKTs expressing a second generation GD2-specific chimeric antigen receptor administered to patients with relapsed or refractory neuroblastoma.
NCT03721068	Study of CAR T-Cells targeting the GD2 with IL-15+iCaspase9 for relapsed/refractory neuroblastoma	I	2018	18	Recruiting	To establish the number of participants with adverse events as a measure of safety and tolerability of iC9.GD2.CAR.IL-15 T cells administered to pediatric subjects with relapsed or refractory neuroblastoma
NCT03635632	C7R-GD2.CART cells for patients with relapsed or refractory Neuroblastoma and other GD2 positive cancers (GAIL-N)	I	2018	94	Recruiting	To determine maximum tolerated dose of C7R-GD2.CART Cells and toxicity.
NCT03618381	EGFR806 CAR T cell immunotherapy for recurrent/refractory solid tumors in children and young adults	I	2018	36	Recruiting	(1) To estimate the maximum tolerated dose and dose limiting toxicities, and describe the full toxicity profile of the two CAR T cell products (2) The number of successfully manufactured EGFR806 and EGFR806xCD19 CAR T cell products will be assessed (3) To establish the safety, defined by adverse events, of EGFR806-specific CAR T cell infusions and of dual transduced EGFR806xCD19 CAR T cell infusions
NCT04483778	B7H3 CAR T cell immunotherapy for recurrent/refractory solid tumors in children and young adults	I	2020	68	Recruiting	(1) To assess the safety and tolerability of cellular immunotherapy utilizing ex-vivo expanded autologous T cells genetically modified to express B7H3-specific CAR (2) To assess the safety and tolerability of cellular immunotherapy utilizing ex vivo expanded autologous T cells genetically modified to express a bispecific B7H3xCD19 CAR. (3) To determine the maximum tolerated dose of B7H3-specific CAR. (4) To determine the maximum tolerated dose of bispecific B7H3xCD19 CAR. (5) To assess the dose limiting toxicities and describe the full toxicity profile for each study arm type, frequency, severity and duration of adverse events will be tabulated and summarized. (6) To assess the feasibility of manufacturing B7H3 specific CARs from patient-derived lymphocytes. (7) To assess the feasibility of manufacturing B7H3xCD19 bispecific CARs from patient-derived lymphocytes.
NCT04637503	4SCAR-T therapy targeting GD2, PSMA and CD276 for treating neuroblastoma	I/II	2020	100	Recruiting	To determine the number of patients with adverse events and the toxicity profile
NCT04539366	Testing a new immune cell therapy, GD2-targeted modified T-cells (GD2CART), in children, adolescents, and young adults with relapsed/refractory osteosarcoma and neuroblastoma, the GD2-CAR PERSIST trial	I	2020	67	Not yet recruiting	To define (i) feasibility of producing GD2-CAR-expressing autologous T-lymphocytes (GD2CART), (ii) incidence of adverse events and (iii) maximum tolerated dose and best response to GD2CART cells

Abbreviations: CAR T, Chimeric Antigen Receptor T cells.

## Data Availability

Data sharing not applicable.
